# ALZHEIMER’S DISEASE AND COMORBIDITIES: A COMPLEX INTERPLAY IN THE CONTEXT OF AGING

**DOI:** 10.1093/geroni/igac059.192

**Published:** 2022-12-20

**Authors:** Konstantin Arbeev, Olivia Bagley, Arseniy Yashkin, Hongzhe Duan, Vinit Nalawade, Igor Akushevich, Svetlana Ukraintseva, Anatoliy Yashin

**Affiliations:** Duke University, Durham, North Carolina, United States; Duke University, Durham, North Carolina, United States; Duke University, Durham, North Carolina, United States; Duke University, Durham, North Carolina, United States; Duke University, Durham, North Carolina, United States; Duke University, Durham, North Carolina, United States; Duke University, Durham, North Carolina, United States; Duke University, Durham, North Carolina, United States

## Abstract

There is evidence on high prevalence of comorbidity in people with dementia and on associations between comorbidities and progression of Alzheimer’s disease (AD). Comorbidities accumulate with age and age is also a major risk factor for AD. Repeated measurements of comorbidity provide possibilities for gaining more knowledge about dynamic interconnection between comorbidities and AD development in the context of aging. We constructed the comorbidity index (CMI) for participants of the Health and Retirement Study aged 66+ years using data on onset of diseases from linked Medicare service use files (6,830 participants, 3,829 females, 3,001 males). We performed the joint analysis of longitudinal measurements of CMI and data on onset of AD and survival since onset of AD using the approach (the stochastic process model) that allows decomposing the overall association of trajectories of CMI with respective time-to-event outcomes into several aging-related characteristics represented by the model’s components and evaluated indirectly from the data. We found that, overall, CMI is significantly (p< 0.0001) associated with increased risk of onset of AD and decreased survival chances for persons with AD and that this association can be decomposed into associations of AD outcomes with different aging-related components with differentiated impact of genetic and non-genetic factors (such as APOE, polygenic scores, sex, birth cohort). In particular, age patterns and time trends in such components contribute to trends in AD prevalence so that taking into account the age dynamics and time trends in comorbidities (represented by CMI) is essential for forecasting future trends in AD prevalence.

